# Correlation of CT features of lung adenocarcinoma with sex and age

**DOI:** 10.1038/s41598-024-64335-7

**Published:** 2024-06-11

**Authors:** Yanli Yang, Yiyi Gao, Fang Lu, Ernuo Wang, Haiquan Liu

**Affiliations:** Department of Radiology, Huadong Hospital, Fudan University, Shanghai, 200040 China

**Keywords:** Lung adenocarcinoma, CT feature, pSD, Sex, Cancer, Lung cancer

## Abstract

This study aimed to retrospectively examine the computed tomography (CT) features of lung adenocarcinoma across different demographic groups. Preoperative chest CT findings from 1266 surgically resected lung adenocarcinoma cases were retrospectively analyzed. Lung adenocarcinomas were categorized based on CT characteristics into pure ground glass (pGGO), nodule-containing ground glass opacity (mGGO), and pure solid without containing ground glass opacity (pSD). These categories were correlated with sex, age, EGFR status, and five histopathological subtypes. The diameters of pGGO, mGGO, and pSD significantly increased across all patient groups (*P* < 0.05). Males exhibited a significantly higher proportion of pSD than females (*P* = 0.002). The mean diameters of pGGO and pSD were significantly larger in males than in females (*P* = 0.0017 and *P* = 0.043, respectively). The frequency of pGGO was higher in the younger age group (≤ 60 years) compared to the older group (> 60 years) for both males (*P* = 0.002) and females (*P* = 0.027). The frequency of pSD was higher in the older age group for both sexes. A linear correlation between age and diameter was observed in the entire cohort as well as in the male and female groups (*P* < 0.0001 for all groups). EGFR mutations were less frequent in pSD compared to pGGO (*P* = 0.0002) and mGGO (*P* < 0.0001). The frequency of lesions containing micropapillary components increased from pGGO to mGGO and pSD (*P* < 0.0001 for all). The frequency of lesions containing solid components also increased from pGGO to mGGO and pSD (*P* = 0.045, *P* < 0.0001, and *P* < 0.0001, respectively). The CT features of lung adenocarcinoma exhibit differences across genders and age groups. Male gender and older age are risk factors for lung adenocarcinoma growth.

## Introduction

Lung cancer is the leading cause of cancer-related death worldwide^1^. It is divided into small-cell lung cancer and non-small-cell lung cancer (NSCLC). NSCLC accounts for 85% of all lung cancers, with adenocarcinoma being the most common histological subtype, comprising 40% of all NSCLC cases^2^. Advancements in molecular biology have significantly improved the prognosis of NSCLC for patients with druggable mutations in the epidermal growth factor receptor (EGFR) or immune checkpoints PD-L1/PD1^3^. The advent of molecular targeted therapy has achieved significant success^4–7^. At the same time, it is essential to understand lung cancer to broaden our perspective comprehensively.

Pulmonary nodules with ground-glass opacity (GGO), defined as hazy lesions on high-resolution CT that do not obscure bronchial structures or pulmonary vessels^8^, have been increasingly observed. This increase is attributable to the growing use of CT in lung cancer screening^9^ and the increased availability of CT technology^10^, leading to a major concern due to increased detection rates^11^.

Persistent pulmonary GGOs are closely associated with lung adenocarcinoma^12^, and various types of GGO in lung adenocarcinoma are associated with different prognoses^13^. The formation of different GGOs has been correlated with factors such as age, sex, smoking habits, and EGFR status^10,14–16^. Previous studies primarily focused on prospective follow-up scenarios. This study aims to examine the influence of sex and age on GGO growth from a clinical perspective.

## Methods

### Patients

The Ethics Review Board of Huadong Hospital, affiliated with Fudan University, approved this retrospective study (No. 20230108) following the Declaration of Helsinki. As this study was retrospective and patient-sensitive information was anonymized, the Institutional Review Committee of Huadong Hospital waived the need for informed consent. Initially, 2,000 patients were consecutively included in this study from January 2018 to December 2022.

### Inclusion and exclusion criteria

The inclusion criteria for the study were patients who (a) underwent surgical resection and (b) were pathologically diagnosed with lung adenocarcinoma. A total of 734 patients were excluded for the following reasons: (a) unavailability of preoperative CT images (n = 56), (b) presence of multiple lesions (n = 350), (c) manifestation of diffuse miliary nodules in both lungs (n = 23), (d) obscuration of lesions by atelectasis, pneumonia, or massive pleural effusion (n = 34), (e) lack of detailed smoking history (n = 113). Additionally, 158 patients with a smoking history were excluded (156 males and 2 females). Therefore, 1266 patients, all of whom were Asian, were included in this study.

### Patients’ general characteristics

Medical records were reviewed to obtain information on sex, age, and smoking history.

### Computed tomographic assessment

Preoperative chest CTs were performed using three scanners: GE Discovery CT750 HD, 64-slice LightSpeed VCT (GE Medical Systems), and somatom definition flash. The scanning parameters were set at 120 kVp, 100–200 mAs, with a pitch of 0.75–1.5 and collimation of 1–1.25 mm. All imaging data were reconstructed using a medium sharp reconstruction algorithm, with a slice thickness of 1–1.25 mm.

### Computed tomographic image interpretation

The CT images were independently interpreted by a clinical radiologist with 35 years of experience (H.Q.L) and two other radiologists, each with 10 years of experience (F.L and E.N.W), on a retrospective basis. The radiologists were blinded to clinical and smoking status. The majority class was used as the final CT feature value. CT images were interpreted using both mediastinal (width = 350 HU; level = 40 HU) and lung (width = 1500 HU; level = −650 HU) window settings. Images were evaluated for lesion size, nodule types, and the presence of single or multiple lesions in both lungs.

Lesion classification was based on ground-glass opacity presence: lesions exhibiting only ground-glass opacity were classified as pure ground-glass opacity (pGGO). Lesions with > 50% but < 100% ground-glass opacity were classified as ground-glass opacity predominant lesions (mGGO). Those with > 1% but ≤ 50% ground glass opacity were classified as solid predominant lesions (mSD). Lesions without ground glass opacity were classified as pure solid lesions (pSD). For statistical purposes, mGGO and mSD were combined and classified as mGGO, resulting in three categories: pGGO, mGGO, and pSD.

### Histopathologic analysis

Lung adenocarcinoma tumors were identified and classified based on the 2011 IASLC/ATS/ERS classification system^17^. Each lesion’s histological subtype (acinar, lepidic, papillary, micropapillary, and solid) was also assessed.

### EGFR mutation status analysis

The mutation status of EGFR exon 18–21 was determined using a polymerase chain reaction-based amplified refractory mutation system (ARMS) with a human EGFR gene mutation fluorescence polymerase chain reaction diagnostic kit (AmoyDx, China) from histology specimens of lung cancer patients.

### Statistical analysis

Statistical analysis was conducted using SAS 9.4 (SAS Institute Inc, Cary, NC). The χ2 test was employed to evaluate differences in the distribution of categorical variables, while an independent t-test was used for continuous variables. Differences were considered statistically significant at *P* < 0.05. A linear correlation was performed between age and tumor diameter in different groups.

## Results

### Patients

The study included 1266 patients, with a mean age of 63.05 years (range 22–90). Of these, 513 were male (mean age 64.04 years, range 24–85), and 753 were female (mean age 62.39 years, range 22–90). All participants were non-smokers.

### The frequency of pGGO, mGGO, and pSD in all populations and different sexes, as well as the diameter of each type of nodule

The diameters of pGGO, mGGO, and pSD consistently increased across all patients, reaching statistical significance (*P* < 0.05) (Table 1). There was no significant difference in the frequency of pGGO between males and females. However, the frequency of pSD was lower in females compared to males (*P* = 0.0002), and the frequencies of mGGO were significantly higher in females than in males (*P* = 0.002) (Table 2). The mean diameters of pGGO and pSD were significantly larger in males than in females (*P* = 0.0017 and *P* = 0.043, respectively) (Table 3).

**Table 1:** Comparison of diameter for different nodules.

	Number	Average	Range	P value
pGGO	389	1.46 ± 0.61	0.53–4.64	
mGGO	367	1.76 ± 0.82	0.5–5.29	< 0.0001
pSD	510	2.17 ± 1.13	0.43–9.50	0.016
Total	1266			

Data for diameter are mean ± standard deviation (cm). Length unit = cm.

*pGGO* pure ground glass opacity nodules, *mGGO* nodules containing GGO, *pSD* pure solid nodules without GGO.

The *P* value was from the diameter of the same row versus the first row.

**Table 2:** Constitute of pGGO, mGGO, and pSD in male and female.

	Male + female (%)	Male (%)	Female (%)	*P* value
pGGO	389 (30.73)	155 (30.21)	234 (31.08)	0.744
mGGO	367 (28.99)	119 (23.20)	248 (32.93)	0.0002
pSD	510 (40.28)	239 (46.59)	271 (35.99)	0.0002
Total	1266	513	753	

Numbers in parentheses are percentages of the same group.

*pGGO* pure ground glass opacity nodules, *mGGO* nodules containing GGO, *pSD* pure solid nodule.

*P* values were from pGGO, mGGO, or pSD compared to other types.

**Table 3:** The diameter comparison of different groups of nodules between male and female.

	Male	Range	Average	Female	Range	Average	*P* value
pGGO	155	0.58–4.64	1.55 ± 0.66	234	0.53–4.08	1.40 ± 0.59	0.017
mGGO	119	0.59–3.97	1.81 ± 0.76	248	0.5–5.29	1.74 ± 0.85	0.442
pSD	239	0.43–9.5	2.27 ± 1.19	271	0.58–6.86	2.07 ± 1.06	0.043

Data for diameter are mean ± standard deviation (cm). Length unit = cm.

*pGGO* pure ground glass opacity nodules, *mGGO* nodules containing GGO, *pSD* pure solid nodules without GGO.

### The constitute of different nodules between ≤ 60 and > 60 in male and female and mean diameter in different group

pGGO was more frequent in the ≤ 60 years group than in the > 60 years group in both male and female (*P* = 0.002 and *P* = 0.026, respectively). Conversely, pSD was more frequent in the > 60 years group, with this difference reaching statistical significance in males and a marginal significance in females (*P* = 0.0138 and *P* = 0.053) (Table 4). The mean diameter of pGGO in patients aged > 60 years was larger than in those aged ≤ 60 years for both sexes, with statistical significance (*P* = 0.0116 for males and *P* = 0.0193 for females) (Table 5). The mean diameters of mGGO and pSD were larger in patients aged > 60 years than in those aged ≤ 60 years for both sexes. Specifically, in males, the diameter of mGGO reached statistical significance (*P* = 0.0357), while in females, it was marginally significant (*P* = 0.0745) (Table 6). However, differences in pSD diameters did not reach statistical significance (Table 7). Linear correlation analyses were performed between age and diameter across the whole group and within male and female subgroups. These analyses showed a linear correlation in all groups (R = 0.189 for the whole group, 0.187 for males, and 0.185 for females, respectively, *P* < 0.0001 for all three groups) (Table 8).

**Table 4:** Constitute of nodules between ≤ 60 and > 60 in male and female.

Male	Male			*P* value	Female			*P* value
	Number	≤ 60 (%)	> 60 (%)		Number	≤ 60 (%)	> 60 (%)	
pGGO	155	67 (38.95)	88 (25.81)	0.002	234	107 (35.67)	127 (28.04)	0.027
mGGO	119	38 (22.09)	81 (23.75)	0.674	248	103 (34.33)	145 (32.01)	0.506
pSD	239	67 (38.95)	172 (50.44)	0.014	271	90 (30.00)	181 (39.96)	0.053
Total	513	172	341		753	300	453	

Data for diameter are mean ± standard deviation (cm). Length unit = cm.

*pGGO* pure ground glass opacity nodules, *mGGO* nodules containing GGO, *pSD* pure solid nodules without GGO.

**Table 5:** The diameter comparison of pGGO between ≤ 60 and > 60 in male and female.

pGGO	Male			Female		
	≤ 60 (n = 67)	> 60 (n = 88)	*P* value	≤ 60 (n = 107)	> 60 (n = 117)	*P* value
Range	0.65–3.02	0.58–4.64		0.53–4.80	0.59–3.94	
Average	1.39 ± 0.52	1.67 ± 0.76	0.012	1.31 ± 0.55	1.48 ± 0.57	0.019

Data for diameter are mean ± standard deviation (cm). Length unit = cm.

*pGGO* pure ground-glass opacity nodules.

**Table 6:** The diameter comparison of mGGO between ≤ 60 and > 60 in male and female.

	Male			Female		
	≤ 60 (n = 38)	> 60 (n = 82)	*P* value	≤ 60 (n = 103)	> 60 (n = 145)	*P* value
Range	0.66–3.42	0.59–3.97		0.50–4.62	0.60–5.29	
Average	1.59 ± 0.66	1.91 ± 0.81	0.036	1.63 ± 0.76	1.82 ± 0.91	0.075

Data for diameter are mean ± standard deviation (cm). Length unit = cm.

*mGGO* nodules containing GGO.

**Table 7:** diameter of pSD of ≤ 60 and > 60 in male and female.

	Male			Female		
	≤ 60 (n = 67)	> 60 (n = 172)	*P* value	≤ 60 (n = 90)	> 60 (n = 181)	*P* value
Range	0.68–5.23	0.43–9.50		0.58–5.16	0.59–6.86	
Average	2.16 ± 1.09	2.32 ± 1.23	0.339	1.90 ± 1.05	2.15 ± 1.06	0.068

Data for diameter are mean ± standard deviation (cm). Length unit = cm.

*pSD* pure solid nodule without GGO.

**Table 8:** Linear correlation between age and diameter of lung adenocarcinoma.

	n	Range in age	Range in diameter	Average age		Average diameter		R	*P* value
Total	1266	22–90	0.43–9.5	63.01	± 10.31	1.83	± 0.96	0.189	< 0.0001
Male	513	24–85	0.43–9.5	64.00	± 10.32	1.95	± 1.02	0.187	< 0.0001
Female	753	22–90	0.50–6.86	62.36	± 1.03	1.75	± 0.90	0.185	< 0.0001

R = correlation coefficient.

### The frequency of EGFR mutation among pGGO, mGGO and pSD

No significant difference was observed in EGFR mutation frequency between pGGO and mGGO (*P* = 0.23). However, the EGFR mutation rates in pGGO [281/389 (72.2%)] and mGGO [279/367 (75.7%)] were significantly higher than those in pSD [308/510 (60.4%)] (*P* = 0.0002 and *P* < 0.0001, respectively) (Table 9).

**Table 9:** EGFR status in different type nodules of lung adenocarcinoma.

	pGGO	mSD	pSD	*P1*	*P2*	*P3*
EGFR mutation	281	379	308	0.23	0.0002	< 0.0001
EGFR wild-type	108	88	202			

*P1*, pGGO versus mGGO, *P2* pGGO versus pSD. *P3*, mGGO versus pSD.

*pGGO* pure ground glass opacity nodules, *mGGO* nodules containing GGO, solid predominant nodules, *pSD* pure solid nodule without GGO.

### Correlation between different nodules and histopathological subtypes

For pGGO, mGGO, and pSD, the relationship between different nodules and pathological subtypes of lung adenocarcinoma was analyzed. Lepidic components were more frequent in pGGO [85/389 (21.8%)] and mGGO [56/367 (15.3%)] than in pSD [28/510 (5.5%)] (*P* < 0.0001 for both comparisons). Acinar components were more frequent in pGGO [282/389 (72.5%)] than in pSD [337/510 (66.1%)] (*P* = 0.04), but differences between pGGO and mGGO or mGGO and pSD were not significant (*P* = 0.22 and 0.47, respectively). There were no significant differences in papillary components among pGGO, mGGO, and pSD (*P* = 0.06, 0.5, and 0.19, respectively). Micropapillary components increased in frequency from pGGO [38/389 (9.8%)] to mGGO [81/367 (22.1%)] and pSD [219/510 (42.9%)] (*P* < 0.0001 for all comparisons). Similarly, solid components increased from pGGO [14/389 (3.6%)] to mGGO [25/367 (6.8%)] and pSD [146/510 (28.6%)] (*P* = 0.045, < 0.0001, and < 0.0001, respectively) (Table 10).

**Table 10:** Correlation between different nodules and pathological subtypes of lung adenocarcinoma.

	pGGO	mGGO	pSD	*P1*	*P2*	*P3*
Lepidic						
With	85	56	28			
Without	304	311	482	0.02	< 0.0001	< 0.0001
Acinar						
With	282	251	337			
Without	107	116	173	0.22	0.04	0.47
Papillary						
With	173	138	173	0.06	0.5	0.19
Without	216	229	237	0.06	0.5	0.19
Micropapillary						
With	38	81	219			
Without	351	286	291	< 0.0001	< 0.0001	< 0.0001
Solid						
With	14	25	146			
Without	375	342	364	0.045	< 0.0001	< 0.0001

*P1*, pGGO versus mGGO. *P2*, pGGO versus pSD. *P3*, mGGO versus pSD.

*pGGO*, pure ground glass opacity nodules, *mGGO* nodules containing GGO, solid predominant nodules, *pSD* pure solid nodule without GGO.

## Discussion

Some pGGOs progressively transform into pSDs^18,19^. Studies indicate that a GGO nodule initially increases in size, followed by the appearance and subsequent enlargement of a solid portion within the lesion^20^. Lesions transitioning from pGGO to mGGO or from mGGO to solid exhibit rapid size increases^10,21,22^. The volume doubling time (VDT) for pGGOs is approximately 600 to 900 days; for part-solid GGOs, it ranges from 300 to 450 days^23^; and for solid nodules, it is about 149 days^24^.

The diameters of pGGO, mGGO, and pSD consistently increased, all reaching statistical significance, which aligns with previous findings. Mets OM et al. also observed that part-solid lesions are larger than pGGOs^25^. Further analysis showed that from pGGO to mGGO and pSD, the frequency of lesions containing micropapillary or solid histopathological subtypes significantly increased. Micropapillary and solid subtypes are highly invasive and associated with poor prognosis in lung adenocarcinoma^26^, explaining the gradual increase in lesion diameters from pGGO to mGGO and pSD. The analysis of EGFR status among pGGO, mGGO, and pSD revealed that the frequency of EGFR wild type was significantly higher in pSD than in pGGO and mGGO, consistent with previous studies^27^.

Previous research has identified male sex as an independent risk factor for GGO growth^14^. In this study, the frequency of pSD was higher and mGGO lower in males compared to females. The transition rate from pGGO through mGGO to pSD appears to be faster in males. Additionally, the mean diameters of pGGO, mGGO, and pSD were larger in males than in females, although the difference in mGGO did not reach statistical significance. These findings suggest a faster growth rate of GGOs in males.

Older age has also been identified as a risk factor for GGO growth^10,25,28^. Using 60 years as the cutoff point, our findings revealed that pGGO was less frequent and pSD more frequent in the > 60 age group, regardless of sex. The rapid growth of pSD compared to pGGO aligns with previous findings. Sui Q et al. analyzed 7605 patients with adenocarcinoma from the SEER (Surveillance, Epidemiology, and End Results) database and concluded that female sex and age ≤ 60 years are independent prognostic factors for better survival, supporting these findings^29^. These results were also confirmed by a larger study involving 61,928 patients conducted by Wu Y et al.^30^.

Furthermore, the diameters of different types of nodules across two age groups were compared. The diameters of pGGO and mGGO in the > 60 group were larger than in the ≤ 60 group (*P* < 0.05), except for the mGGO group in females, where the difference did not reach statistical significance (*P* = 0.0745). For pSD, there was no statistically significant difference in diameter between the two age groups for both sexes. A linear correlation analysis between age and nodule diameter for all groups (*P* < 0.0001 for all) further supports the conclusion that older age is a risk factor for tumor growth.

This study compared risk factors in sex and different ages for lung adenocarcinoma, excluding patients with a smoking history, which might reflect differences under natural conditions and provide a reference for future research. This study further affirms that lung adenocarcinoma is highly personalized^31–33^. In the future, a precise model for evaluating lung adenocarcinoma is needed.

In conclusion, being male and older age are risk factors for GGO growth. However, this study had several limitations: (1) The pathology of the disease was not included, and lung adenocarcinoma progression was evaluated solely through CT. (2) Due to its retrospective nature, it was not possible to calculate the VDT of GGO nodules; instead, diameter and GGO types were used as alternatives. (3) The assessment of GGO proportions was based on visual evaluations without quantitative analysis of CT values. (4) This study did not distinguish whether patients were seen symptomatically or discovered during a physical examination. Future studies will address these aspects.

## Data Availability

The data that support the findings of this study are not openly available due to reasons of sensitivity and are available from the corresponding author upon reasonable request.

## Abbreviations

pGGO: Pure ground glass opacity nodules
mGGO: Ground glass opacity predominant nodules
mSD: Solid predominant nodules
pSD: Pure solid nodule
CT: Computed tomography
